# Biobanking in a Challenging African Environment: Unique Experience from the SIREN Project

**DOI:** 10.1089/bio.2017.0113

**Published:** 2018-06-01

**Authors:** Rufus O. Akinyemi, Kazeem Akinwande, Samuel Diala, Osi Adeleye, Abiodun Ajose, Kehinde Issa, Dorcas Owusu, Isaac Boamah, Isah Suleiman Yahaya, Abdulraheem Olayemi Jimoh, Lucius Imoh, Gregory Fakunle, Albert Akpalu, Fred Sarfo, Kolawole Wahab, Emmanuel Sanya, Lukman Owolabi, Reginald Obiako, Godwin Osaigbovo, Morenikeji Komolafe, Michael Fawale, Philip Adebayo, Paul Olowoyo, Yahaya Obiabo, Taofiki Sunmonu, Ijezie Chukwuonye, Olayemi Balogun, Basirat Adeoye, Florence Oladele, Peter Olowoniyi, Frederick Adeyemi, Arthur Lezzi, Ajibola Tunde Falayi, Michael Fasanya, Kolawole Ogunwale, Olabisi Adeola, Omolara Olomu, Olumayowa Aridegbe, Ruth Laryea, Ezinne Uvere, Moyinoluwalogo Faniyan, Ezinne Melikam, Raelle Tagge, Onoja Akpa, Joshua Akinyemi, Oyedunni Arulogun, Hemant K. Tiwari, Bruce Ovbiagele, Mayowa O. Owolabi

**Affiliations:** ^1^ Center for Genomic and Precision Medicine, College of Medicine, University of Ibadan, Ibadan, Nigeria.; ^2^ Neuroscience and Ageing Research Unit, Institute for Advanced Medical Research and Training, College of Medicine, University of Ibadan, Ibadan, Nigeria.; ^3^ Pathology Department, Federal Medical Centre, Abeokuta, Nigeria.; ^4^ Neurology Unit, Department of Medicine, College of Medicine, University of Ibadan, Nigeria.; ^5^ Neurology Unit, Department of Medicine, Obafemi Awolowo University Teaching Hospital, Ile-Ife, Nigeria.; ^6^ Neurology Unit, Department of Medicine, Kwame Nkrumah University of Science & Technology, Kumasi, Ghana.; ^7^ Neurology Unit, Department of Medicine, School of Medicine & Dentistry, College of Health Sciences, University of Ghana, Accra, Ghana.; ^8^ Neurology Unit, Department of Medicine, Bayero University, Kano, Nigeria.; ^9^ Neurology Unit, Department of Medicine, University of Ilorin Teaching Hospital, Ilorin, Nigeria.; ^10^ Neurology Unit, Department of Medicine, Jos University Teaching Hospital, Jos, Nigeria.; ^11^ Neurology Unit, Department of Medicine, Ahmadu Bello University Teaching Hospital, Zaria, Nigeria.; ^12^ Neurology Unit, Department of Medicine, Ladoke Akintola University Teaching Hospital, Ogbomoso, Nigeria.; ^13^ Neurology Unit, Department of Medicine, Federal University Teaching Hospital, Ido Ekiti, Nigeria.; ^14^ Neurology Unit, Department of Medicine, Delta State University Teaching Hospital, Igharra, Nigeria.; ^15^ Neurology Unit, Department of Medicine, Federal Medical Center, Owo, Nigeria.; ^16^ Neurology Unit, Department of Medicine, Federal Medical Center, Umuahia, Nigeria.; ^17^ Department of Neurosciences, Medical University of South Carolina, Charleston, South Carolina.; ^18^ School of Public Health, University of Alabama, Birmingham, Alabama.

**Keywords:** biobanking, stroke, neurological disorders, Low and Middle Income Countries (LMIC), Africa, genomics, trans-omics

## Abstract

Africa was previously insufficiently represented in the emerging discipline of biobanking despite commendable early efforts. However, with the Human, Heredity, and Health in Africa (H3Africa) initiative, biorepository science has been bolstered, regional biobanks are springing up, and awareness about biobanks is growing on the continent. The Stroke Investigative Research and Educational Network (SIREN) project is a transnational, multicenter, hospital and community-based study involving over 3000 cases and 3000 controls recruited from 16 sites in Ghana and Nigeria. SIREN aims to explore and unravel the genetic and environmental factors that interact to produce the peculiar phenotypic and clinical characteristics of stroke as seen in people of African ancestry and facilitate the development of new diagnostics, therapeutics, and preventative strategies. The aim of this article is to describe our experience with the development of the procedure for collection, processing, storage, and shipment of biological samples (blood, serum, plasma, buffy coat, red cell concentrates, and DNA) and brain imaging across coordinating and participating sites within the SIREN Project. The SIREN network was initiated in 2014 with support and funding from the H3Africa Initiative. The SIREN Biobank currently has 3015 brain images, 92,950 blood fractions (serum, plasma, red cell concentrates, and buffy coat) accrued from 8450 recruited subjects, and quantified and aliquoted good-quality DNA extracts from 6150 study subjects. This represents an invaluable resource for future research with expanding genomic and trans-omic technologies. This will facilitate the involvement of indigenous African samples in cutting-edge stroke genomics and trans-omics research. It is, however, critical to effectively engage African stroke patients and community members who have contributed precious biological materials to the SIREN Biobank to generate appropriate evidence base for dealing with ethical, legal, and social issues of privacy, autonomy, identifiability, biorights, governance issues, and public understanding of stroke biobanking in the context of unique African culture, language, and belief systems.

## Introduction

Biobanking is a rapidly evolving field of biomedical science that involves the collection, processing, storage, and distribution of biospecimens and the policies and procedures involved.^1^ Although initially focused on collecting samples for diagnostic purposes in pathology settings, biobanks have since evolved into organized repositories and networks that are now engaged in cutting-edge translational research which form the bedrock of personalized (or precision) medicine.^2,3^ Furthermore, this evolution has involved the development of biobanking best practices and the transformation of the field into the emerging field of biospecimen science.^4,5^

In the past, Africa was underrepresented in the biobanking revolution with the few available biobanks serving mainly as conduits of samples to the developed countries.^6^ Early biobanking efforts include those of the MalariaGen Network^7^ and those reported in the Low and Middle Income Countries Biobank and Cohort Network (BCNet).^8^ However, with the Human, Heredity, and Health in Africa (H3Africa) initiative,^9^ biobank science has been bolstered, regional biobanks are springing up, and awareness about biobanks is growing on the continent.^10–12^

Following the revolutionary sequencing of the human genome in 2003 and the subsequent development of genetic and genomic technologies, disease-specific consortia bringing together many individual sites and collaborators have evolved for many major diseases, including stroke.^13,14^ Sub-Saharan African countries contribute significantly to the global burden of stroke, with the highest age-standardized incidence rate (316/100,000) and prevalence (1460/100,000). Furthermore, African countries together with all LMIC suffer up to 86% of all stroke-related deaths around the world.^15,16^

The Stroke Investigative Research and Educational Network (SIREN) project is a transnational, multicenter, hospital and community-based study involving 3000 cases and 3000 controls recruited from 16 sites in Ghana and Nigeria. SIREN is poised to identify unique genetic and nongenetic factors associated with stroke occurrence, type, subtype, pattern, and outcomes in indigenous Africans. This project aims to explore and unravel the genetic and environmental factors that interact to produce the peculiar phenotypic and clinical characteristics of stroke as seen in people of African ancestry and facilitate the development of new diagnostics, therapeutics, and preventative strategies.^17^

The aim of this article is to describe our experience with the development of efficient and reliable procedures for collection, processing, storage and shipment of biological samples (blood, serum, plasma, buffy coat, red cell concentrates, and DNA) and brain images across coordinating and participating sites within the SIREN Project, despite infrastructural challenges common in LMIC settings. We present a model with lessons that can be useful for other biorepositories springing up in LMICs with similar challenges.

## Development of the SIREN Biobank

Blood samples were obtained from all SIREN study participants (cases and controls), separated into serum, plasma, red blood cell concentrates, and buffy coats for subsequent analysis to find novel and emerging biomarkers of stroke, as well as extracting DNA for genomic studies. These fractions, however, needed to be stored in the biorepository to maintain sample integrity and stability for the crucial analyses for stroke genes and biomarkers.

SIREN is a case–control study involving several collaborating centers in Nigeria and Ghana (Table 1) with the coordinating center being the University of Ibadan/University College Hospital Ibadan.^17,18^

**Table 1.** Characteristics of SIREN Study Sites and Their Biorepository Facilities

*Site*	*Dominant ethnic groups*	*Description of hospital site*
Korle Bu Teaching Hospital, Accra, Ghana	Akan	This is a 1500-bed tertiary hospital serving as the main referral hospital of the southern part of Ghana, and of the capital Accra. Stroke is the leading cause of admissions to the medical wards (up to 30 cases per month) of the KBTH with a case fatality rate (up to 22%). There is currently an ongoing collaboration to improve stroke care in the Wessex Ghana stroke project. Two sets of −20°C freezers are available, a −80°C freezer is outsourced, and they rely on the hospital's backup power system.
	Ewe	
	Ga/Adangbe	
Komfo Anokye Teaching Hospital, Kumasi, Ghana	Akan	This is the second largest hospital in the country and the only tertiary health institution in the Ashanti Region. It is the main referral hospital for the Ashanti, Brong Ahafo, Northern, Upper East, and Upper West Regions. It is the teaching hospital affiliated to the medical school of the Kwame Nkrumah University of Science and Technology. The hospital currently has about 1000 beds up from the initial 500 when first built. Stroke also forms a major cause of admission (48 cases per month) and with mortality of up to 30%_.._ Two sets of −20°C freezers are available, a −80°C freezer is outsourced, and they rely on the hospital's backup power system.
	Ewe	
	Ga/Adangbe	
University College Hospital and Blossom Center for NeuroRehabilitation, Ibadan, Nigeria	Yoruba	UCH has 850 bed spaces and 163 examination couches. The hospital is a tertiary institution with several affiliated community care centers, where the hospital offers secondary and primary healthcare. Four −80°C freezers and three −30°C freezers are available, all backed up by hybrid solar-powered inverter systems installed by SIREN. Up to 18 cases of stroke are seen per month with mortality of about 28%.
		Blossom Center for NeuroRehabilitation, was established in 2010 through the support of the World Federation for Neurorehabilitation as the first center for Neurorehabilitation in the East, West, and Central Africa. A −20°C freezer is available, as well as an inverter system both installed by SIREN.
Federal Medical Center, Abeokuta, Nigeria and Sacred Heart Hospital, Abeokuta	Yoruba	FMC is a 250-bed regional tertiary center established in April 1993. It receives patients from Ogun and neighboring states and countries, and relates closely with community care clinics within and outside the Abeokuta metropolis. Sacred Heart Hospital is the oldest missionary hospital in Nigeria and serves the lower rungs of the society. Two sets of −20°C freezers are available (1 per site) as well as two power backup inverter systems, both installed by SIREN. About 12 cases of stroke are seen per month with a mortality of 20%.
Aminu Kano Teaching Hospital (AKTH), Kano-Nigeria	Hausa	The hospital is a tertiary referral health center established in 1988 for Kano state and its neighboring states, such as Jigawa, Katsina, Zamfara, Bauchi, Gombe, and Yobe. A −40°C freezer is available as well as an inverter system, both installed by SIREN. Up to 16 cases of stroke are admitted per month with a high mortality of up to 38%.
Ahmadu Bello University Teaching Hospital, Zaria, Nigeria	Hausa	It is located in Zaria, Northern Nigeria, and consists of three sites located in Kaduna, and Zaria (in Kaduna State), and Malumfashi (in Katsina State), with a total bed capacity of 768. It is still a major referral center for the 19 Northern States, but receives patients predominantly from the Northwestern States of Kaduna, Katsina, Zamfara, and Kano; and adjoining Northcentral States of Niger, Kogi, and Nassarawa, including Abuja, the Federal Capital. A −40°C freezer is available as well as an inverter system both installed by SIREN. Zaria site also outsourced 4 additional freezers. About 25–30 cases of stroke are admitted per month and mortality rate is up to 30%.
University of Ilorin Teaching Hospital, Ilorin, Nigeria	Yoruba	UITH is a 550-bed hospital with 130 beds for medical inpatients, of which 10 are dedicated to acute stroke care. This is in addition to 23 beds for medical emergencies. Between 20 and 30 stroke cases are admitted per month and most of these are managed in a dedicated Acute Stroke Care Unit. Mortality rate is about 20%. The hospital is equipped with CT and MRI scanners, whereas carotid Doppler studies and cardiac Holter monitoring are also available for patients who require these. In addition, the hospital provides community-based primary healthcare services in 3 adjoining states, namely, Esie in Kwara, Ihima in Kogi, and Kishi in Oyo states. A −40°C freezer is available as well as a hybrid power backup inverter system both installed by SIREN.
Obafemi Awolowo University Teaching Hospitals Complex, Ile-Ife, Nigeria.	Yoruba	Obafemi Awolowo University Teaching Hospitals Complex (OAUTHC), established in 1975, is a 576-bed hospital, which consists of 3 specialist referral hospitals, including Ife Hospital Unit, Ile-Ife (342-Bed Complement), Wesley Guild Hospital, Ilesa (212-Bed Complement), and the Dental Hospital, OAU, Ile-Ife (36 Dental Chairs). The hospital complex also includes two urban and one rural primary healthcare centers located at Ife, Ilesa, and Imesi-Ile, respectively, for the purpose of training of health professionals in primary care. OAUTHC offers acute stroke care and rehabilitation at the Ife Hospital Unit and Wesley Guild Hospital unit. About 10 cases of stroke are 3 seeing per month with a mortality rate of 42%. By virtue of its location and the scarcity of healthcare facilities in neighboring states, OAUTHC has an extensive catchment, which includes the whole of the States of Osun, Ekiti, and Ondo and some parts of the States of Oyo, Kwara, and Kogi.. A −40°C freezer is available provided by SIREN. However, there is no power backup system, the laboratory relies solely on the hospital power supply.
Ladoke Akintola University of Technology Teaching Hospital, Ogbomoso, Nigeria	Yoruba	LAUTECH Teaching Hospital was established in May 2011 as an additional teaching hospital of the medical faculty of LAUTECH, which has its main teaching hospital in Osogbo, Osun State, Nigeria, a city located about 57 km from the new facility. Although a 600-bed space tertiary institution, it is currently using about 300-bed spaces. The hospital serves Ogbomoso populace and other communities from Oyo state and the adjoining states of Osun and Kwara. Acute stroke cases are managed on the medical ward. Three cases of stroke are seen per month with a mortality rate of 33%. Investigations and imaging support are provided by the respective departments in the hospital. A −40°C freezer is available provided by SIREN. However, there is no power backup system, and the laboratory relies solely on the hospital power supply.
Jos University Teaching Hospital, Jos, Nigeria	Hausa	JUTH was established in 1981. It is a 620-bed space tertiary health facility. Apart from the temporary and permanent sites, the hospital also runs comprehensive health centers at zamko and Gindiri, which serves as referral centers to the teaching hospital in Jos. The hospital is located in Lamingo, Jos North and receives between 8 and 14 cases of stroke per week, and these patients are managed in the medical wards unless ICU care is warranted. Mortality rate is about 20%. It has the following facilities: CT and MRI scanners, carotid Doppler study facilities, and a cardiac Holter monitor for patients who may require such investigative modalities. It receives referrals from neighboring states, such as Bauchi, Gombe, Benue, Niger, Kogi, Nassarawa, Taraba, Adamawa, Kaduna, and the Federal capital territory. A −20°C freezer is available provided by SIREN. However, there is no power backup system, and the laboratory relies solely on the hospital power supply.
	Berom	
Delta State University Teaching Hospital, Oghara, Nigeria		DELSUTH is a 300-bed tertiary hospital commissioned in 2010. It is affiliated to the Delta State University, Abraka. The center is equipped with CT and MRI diagnostic facilities serving over 4 million multiethnic people of Delta State. It also serves as the referring center for other neighboring states of Edo, Bayelsa, Ondo, Anambra, and Rivers. Stroke is the leading cause of neurological admissions in the center with 6 cases per month and mortality rate of 25%. The hospital is closely affiliated to secondary centers located in Warri, Eku, Sapele, Ugheli, Agbor, and Asaba; and primary centers in Oghara, Mosogar, Abraka communities for secondary and community-based services. A −20°C Freezer (outsourced) is available.
Federal Teaching Hospital, Ido Ekiti, Nigeria	Yoruba	The Federal Teaching Hospital, Ido Ekiti was established in July 1998 from the former Ido General Hospital. It was formerly known as the Federal Medical Center Ido Ekiti until November 2014, when it was upgraded to a teaching hospital for the training of the medical students of Afe Babalola University, Ado-Ekiti.
		It is situated in Ido-Osi Local Government Area of Ekiti State, located in the southwestern region of Nigeria. Ido Ekiti Community is a semiurban population that consists majorly of small-scale business holders, farmers, and civil servants who are mostly workers at the Teaching Hospital.
		Federal Teaching Hospital Ido Ekiti is a 169-bed center that runs general and specialized inpatients/outpatients clinical activities for people in its catchment area, which covers Ekiti State and adjoining parts of Kwara, Osun, Ondo, and Kogi states. Up to 24 cases of stroke are admitted per month with a mortality rate of 30%.
		A −40°C freezer is available provided by SIREN. However, there is no power backup system, and the laboratory relies solely on the hospital power supply plus generator in the event of conventional power outage.
Federal Medical Center, Owo, Nigeria	Yoruba	The hospital is a 300 bedded hospital in south west Nigeria. It receives patients from about six neighboring states i.e Edo, Kogi, Osun Delta, Ogun, Ekiti, and FCT Abuja. The hospital also has community health centers in Akure, Ijebu Owo, and Ikaramu in Ondo state. Up to 24 cases of stroke are admitted per month with mortality rate of 33%. It has neuroimaging facilities, such as CT scan, and intensive care unit for management of stroke patients and other critically ill patients. A −40°C freezer is available provided by SIREN. However, there is no power backup system, and the laboratory relies solely on the hospital power supply and generator in the absence of conventional power supply.
Federal Medical Center, Umuahia, Nigeria.	Igbo	Federal Medical Center Umuahia, a 405-bed (still counting) tertiary hospital, occupying 77 acres of space. It is one of the leading healthcare providers in south eastern Nigeria. The facility is centrally located and readily accessible from Enugu, Imo, Rivers, Ebonyi, Akwa-Ibom, and Anambra States. The hospital clients and patients are drawn from all over the country, but predominantly from the south–east and south–south regions of the country. Up to 25 cases of stroke are seen per month with a mortality rate of about 20%. The hospital environment is clean, with a welcoming ambiance. A −40°C freezer is available provided by SIREN. However, there is no power backup system, and the laboratory relies solely on the hospital power supply and generator in the event of power outage.

SIREN, Stroke Investigative Research and Educational Network.

Cases are hospital-based patients with first episode of stroke, recruited within 10 days of onset of symptoms and with neurovascular imaging confirmation. Etiological and topographical stroke subtypes are documented for all cases. Controls are hospital- and community-based participants, matched with cases on the basis of gender, ethnicity, and age (±5 years). Information is also collected on known and emerging risk factors. The central coordinating center (the central site), houses the samples and data contained within SIREN biobank. Other collaborating centers referred to as peripheral sites, enroll subjects, collect samples and necessary data, process the samples, and store temporarily before shipment to the central biobank (Table 1).

The SIREN central site at the University College Hospital Ibadan is equipped with biobanking facilities, including three −30°C freezers for short-term storage of specimens (3–4 weeks), four ultra-low temperature −80°C freezers for long-term storage (and access to two backup extra −80°C freezers for temporary emergency storage). Peripheral sites are each equipped with at least one −20°C or −40°C freezer for short-term storage of specimens (3–4 weeks) before shipment to the central site. Some peripheral sites also have access to −80°C freezers (e.g., Kano, Zaria, Kumasi, Accra).

**FIG. 1.**
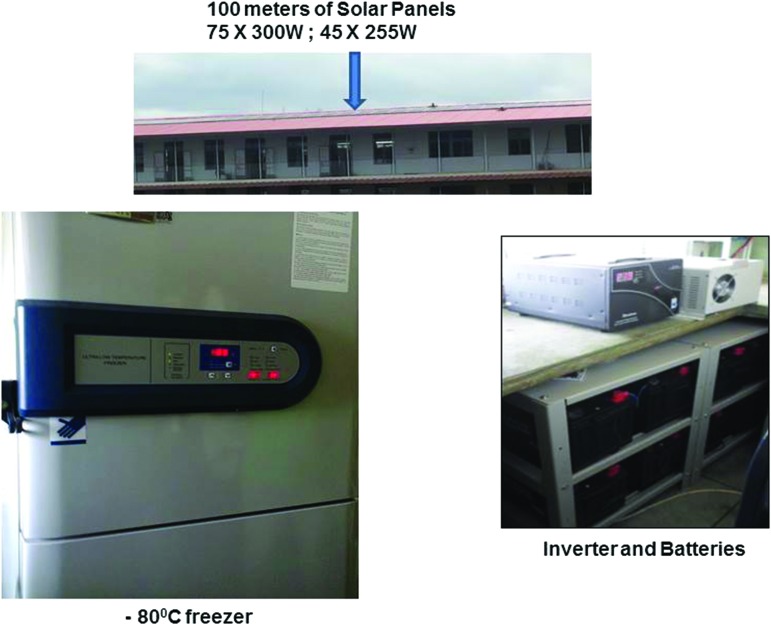
SIREN Backup Solar Power. SIREN, Stroke Investigative Research and Educational Network. Color images available online at www.liebertpub.com/bio

Because of the epileptic nature of electric power supply from the national grid in Nigeria, all facilities are backed up with an alternate power supply using inverter energy storage systems to complement the public energy supply when there is a failure. The biorepository at the central site has solar-powered hybrid inverter batteries providing a backup power source for the ultra-low temperature (−80°C) freezers, whereas freezers at peripheral sites are backed up with electric powered inverters (Fig. 1). Temperature charts are kept for every freezer at all sites with recordings taken every morning and evening on a daily basis by the site research laboratory scientist. These are also forwarded to the central site for archiving. The Freezerworks software (Dataworks Development, Seattle, WA) is employed as a laboratory management information system for labeling and tracking samples within the biorepository.^19^

**FIG. 2.**
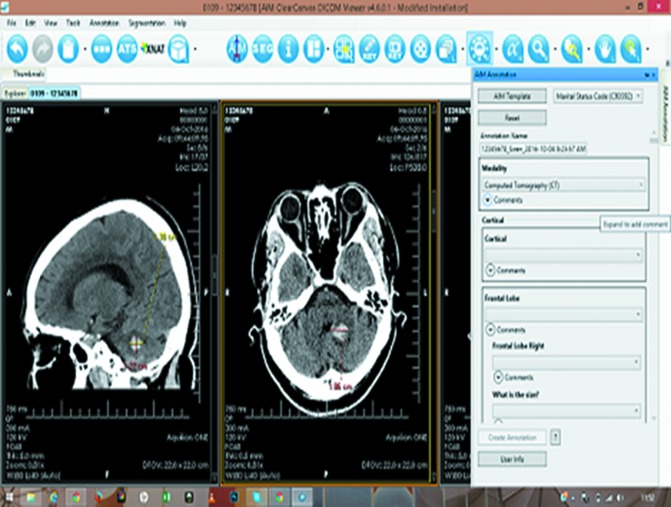
ACCESS Software. Color images available online at www.liebertpub.com/bio

In addition, the SIREN team has developed a neuroimaging databank, the Annotation and Image Markup on Clear Canvas Enriched Stroke–phenotyping Software (ACCESS).^20^ This is a novel standalone computer software application that allows the creation of simple standardized annotations for reporting brain images of all stroke types. The ACCESS application facilitates a concordant and reproducible classification of stroke subtypes by multiple investigators, thus making it suitable for clinical use and multicenter research (Fig. 2). The user-friendly ACCESS software for image analysis has a dedicated secure and backed up multiterabyte server for archiving neuroimages and currently has over 3000 CT/MRI brain scans stored.

## Study Protocols, Approvals, and Material Transfer Agreement

Conforming to international ethical standards in research, ethical approvals were obtained from the Institutional Review Boards of the central site and each participating site. A broad informed consent was also obtained from each participant. A detailed Information Sheet and Consent Form were made available to each participant in the language best understood by the individual.^17^

Standard Operating Procedures (SOPs) were developed for management of biological samples, including blood and blood fractions (serum, plasma, buffy coat, red cell concentrates, and DNA extracts) and urine. There were also specific SOPs developed for sample collection, specimen handling, and transportation to the laboratory, sample processing, sample storage, sample shipment, selected laboratory analysis (measurement of plasma blood glucose, glycosylated hemoglobin, and lipid profiles), extraction of nucleic acid material, and distribution in line with the principles of custodianship for responsible oversight of biospecimens collected for research.^21^

All laboratories in the SIREN performance sites adopted the approved final versions of the SOPs. To ensure compliance with the SOPs, periodic visits were made to the sites and effective communication channels (emails, biweekly teleconferences, and multimedia messaging using WhatsApp) were established among the laboratory scientists working on the SIREN Project. For shipment of biosamples from the peripheral to the central site as well as shipment of DNA samples to the collaborating institution abroad, we developed a material transfer agreement (MTA) in accordance with the guidelines of the Nigerian National Health Research Ethics Committee, the Nigerian Code of Research Ethics, and the H3Africa Data and Biospecimen Access Guidelines.^22^ SIREN, as a member of the H3Africa Consortium, is banking her biospecimens with the H3Africa central biorepository. Accessing these biospecimens follows the guidelines stipulated by the H3Africa Data and Biospecimen Access Committee Guidelines, which are eloquently described in the cited article by Beiswanger et al.^20^ Briefly, a non-H3Africa investigator who meets stipulated criteria can send a request to the H3Africa Secretariat. This request will be forwarded to the Data and Biospecimen Access Committee for necessary considerations. If the request for samples is approved, the requestor and biorepository will coordinate shipment of the samples after signing an MTA.

## SIREN Biorepository and International Best Practices in Biobanking

The International Society for Biological and Environmental Repositories (ISBER) has developed generic benchmarks for internationally accepted best practices in biorepository science,^5^ while the International Stroke Genetics Consortium (ISGC) has also published recommendations for sample management processes and infrastructure necessary for large-scale genetic efforts as it applies to stroke.^13,14^ Despite limited resources, the SIREN Study complied with the ISBER Best Practices and ISGC recommendations (Table 2). The peculiar challenges faced, solutions devised to mitigate the challenges, and lessons learned are discussed in Data and Biospecimen Access and Exploitation Plan section of this article.

**Table 2.** International Best Practices in Biorepository Science and How Met in the SIREN Project

*ISBER and ISGC Best Practices^5,11,12^*	*How met in the SIREN project*
Planning considerations (models, funding, personnel)	Planned with support of H3Africa Biorepository program; Funded by NIH (H3Africa Initiative); Dedicated, trained biorepository staff
Storage Facilities	Dedicated space with freezers (−20°C to −40°C chest freezers, and −80°C ultra-low temperature freezers, refrigerators; −30°C freezers at peripheral sites with inverter power backup
	Solar power energy backup at central sites (75 × 300 W; 45 × 220 W solar panels)
	Adequate lighting, controlled access; fire extinguishers;
Quality Management	Staff schedules; Quality Manuals (Standard Operating Procedures); Quality standards development and validation of sample processing methods and sample quality by I–HAB; fortnightly meetings of SIREN group; Visits to site biorepositories
Safety	Safety protocols; Health and safety training for staff and team members; personal protective wears for staff
Training	SIREN Team biorepository training; staff training at I–HAB; Staff retraining programs
Records Management	Uniform phenotype data collection case report forms; ACCESS Neuroimaging Bank; Freezerworks Laboratory Information Management System (LIMS); REDCap Online data management platform
Cost Management	Procurement adheres to standard operating procedures of H3Africa and University of Ibadan; Rigorous negotiation with shipping companies and other service providers
Biological Material Tracking	Facilitated by Freezerworks LIMS; Continuous engagement with site biorepositories
Packaging and Shipments	Adherence to IATA protocols and standards; synergy with I – HAB
Specimen Collection, Processing, and Retrieval	Blood specimen collected at site as per protocol; separation into blood fractions at site as per protocol; DNA extraction at hubs in Ghana and central site in Ibadan, Nigeria as per protocol; Retrieval and shipment from peripheral and central sites as per protocol
Specimen Access, Utilization, and Destruction	Adherence to H3Africa Data and Biospecimen Access Committee Guidelines.
Ethical, Legal, and Social Issues	Institutional Ethics Committee approval in each participating site and at the central site; approved material transfer agreement; data and samples are deidentified; broad informed consent obtained from study participants; transparent governance, and robust community engagement programs

ISBER, International Society for Biological and Environmental Repositories; ISGC, International Stroke Genetics Consortium.

While setting up the SIREN Biobank, we had access to H3Africa Consortium-wide resources in terms of experienced personnel, training, and other resource materials. We also had direct access to the H3Africa Biorepository Hub at the Institute of Human Virology, Abuja Nigeria. Two staff members from the SIREN team (Lead Biorepository Manager and Laboratory Information Management System [LIMS] Manager) had periods of hands-on training at the H3Africa Biorepository Hub at the Institute of Human Virology, Abuja, Nigeria. The SIREN team has established an excellent working relationship with the H3Africa Biorepository Team at the Institute of Human Virology Abuja, Nigeria (I–HAB) in the process of developing the biobank. There are regular consultations by email and telephone as well as face-to-face meetings during H3Africa consortium meetings. Before the commencement of biobanking activities, personnel from the I–HAB team facilitated a biorepository training workshop organized at the SIREN coordinating site in Ibadan with participating teams from all the SIREN peripheral sites (site principal investigator, laboratory scientist/site biorepository coordinator, and site study coordinator).

## Protocols of the SIREN Biobank

### Informed consent and uniform phenotype data collection

All potential study participants are taken through an informed consent form and those who agree to participate in the study are required to sign two copies (one for SIREN and the other for the study participant to keep). The consent forms are included in the Supplementary Data (Supplementary Data are available online at www.liebertpub.com/bio). Consenting to participate also implied that the subject allows biospecimen storage in the SIREN BioBank. Akpalu et al. described the processes of uniform phenotype data collection from subjects across all sites.^17^ Briefly, patients admitted in participating centers who fulfill the case definition of stroke are logged as case subjects and invited to participate in the study. Those that meet the eligibility criteria and provide informed consent are enrolled into the study after duly completing the informed consent processes. Control subjects are primarily community based, but hospital-based controls, including attendants or relatives of another (nonstroke) patient, or patients admitted or visiting the hospital for conditions not related to stroke are also recruited. Each control subject was matched with a case with respect to age, sex, and ethnicity. Inclusion and exclusion criteria for participation are as previously described.^17^

### Standardized sample collection

At the commencement of the SIREN project, medical laboratory scientists and research assistants across all participating sites were trained on the biorepository processes of the project at the central site in Ibadan, Nigeria. Protocols were developed for participant identification and enrolment as well as SOPs for sample collection (phlebotomy), processing, storage, and immediate analysis of some biomarkers at peripheral sites.^17^ Kits for specimen (blood and urine) collection are centrally procured by the SIREN Central Laboratory (central site) and distributed across all peripheral sites, with the expiration dates of the kits clearly indicated on the kit label. The kits had a shelf life of 6 months and sample containers that would last the defined period were supplied to each site. Expiration dates on the kits (dates before which blood samples must be collected) were also entered into the subjects' case reporting forms (CRF) at recruitment and sample collection. Sample collection kits for individual subjects were packaged in individual plastic bags. The contents of the sample collection kit for each subject are listed in Table 3.

**Table 3.** Blood and Urine Collection Kit Contents

Sample collection
1 set of 14 barcode labels
2 × 5 mL Plain red top (or 1 × 10 mL red) vacutainer collection tube (serum)
1 × 20-mL urine collection container
1 × 10 mL gold-top SST vacutainer collection tube (serum)
1 × 4.5 mL blue-top (Na Citrate) vacutainer collection tube (plasma + cells)
4 × 4.5 mL purple-top (K3EDTA) vacutainer collection tube (plasma, rbc, buffy coat, HbA1c)
1 × 4.5 mL light green (lithium heparin) vacutainer collection tube (plasma lipid profile)
1 vacutainer needle or vacutainer butterfly needle
1 tourniquet (nonlatex)
Sample processing
11 × 1.8-mL cryovial
1 × 0.5-mL cryovial
disposable plastic pipettes/adjustable volume pipettes & disposable pipette tips

Before commencement of sample collection, strips of Freezerworks' barcode labels are applied on tubes and containers requiring labeling (blood collection tubes, urine collection container, and 2-mL storage vials) and the subject's CRF. The labeled storage vials are returned into the resealable plastic bag for later use. For urine sample collection, subjects are asked to provide a spot urine specimen in the large 20-mL urine collection container onto which the label has been applied, ensuring at least 2-mL of urine in the urine collection container.

**FIG. 3.**
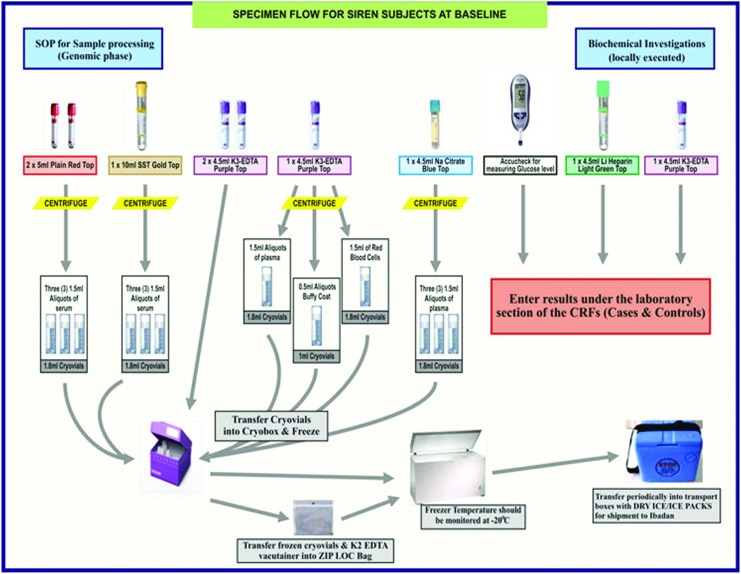
SIREN Sample Workflow. Color images available online at www.liebertpub.com/bio

Blood samples are obtained from each case within 10 days of symptom onset, and from each control upon enrollment after an overnight fast and into relevant anticoagulant-coated or plain tubes as described in the SIREN SOP. The order of sample collection into the collection bottles is also described (Fig. 3 and Supplementary Data). Following collection, collection time and date are clearly indicated while the samples are packed in a nonsealable plastic Ziplock bag. This is then placed in another bag containing adequate packing material (such as tissue paper, cotton wool) to absorb liquid if leakage occurs accidentally. This package is then placed in a small-sized cooler containing ice packs for a maximum of 6 hours to maintain proper cold chain system during transit to the laboratory. The box is sealed securely and clearly labeled as “BIOHAZARD MATERIAL.” Each transport container is accompanied with completed forms showing sample details and relevant subject identification data.

### Sample processing

SIREN peripheral sites use the local laboratory facilities to initially process specimens before temporary storage and shipment to the SIREN central laboratory for relevant analysis and/or long-term storage.

The laboratory is notified ahead of sample collection and sample processing is usually carried out within one hour of sample collection, but not exceeding two hours. Sample processing involves the preanalytical separation of blood samples into different fractions that would be used during subsequent laboratory analyses of such specimen. All blood samples collected are centrifuged at 3000 rpm for 20 minutes (2500 rpm for samples in Na citrate bottles) and separated into relevant fractions (serum, plasma, buffy coat, and red cell concentrates). In situations of delayed processing, samples in collection tubes are stored at 4°C for a maximum of one hour. Table 4 shows further details of the guideline for SIREN sample processing, whereas Figure 3 describes the specimen workflow. It is ensured that all materials needed are in place before sample collection and processing.

**Table 4.** Guideline for SIREN Sample Processing

*Sample*	*Tube*	*Local specimen preparation*	*Received at central laboratory (Ibadan or Accra)*	*Aliquot for storage, in local laboratory*
A (Blood)	2 × 5 mL Serum Plain RED top	Allow to clot for 30 minutes		Aliquots - A1, A2, A3. Store for future testing
		Centrifuge for 20 minutes at 3000 rpm		
		Transfer 1.5 mL of supernatant into 3 × 1.8-mL cryovials		
		Affix barcode label		
		Store supernatant at −20°C		
		Ship on dry ice		
B (Blood)	10 mL SST Gold top	Allow sample to clot for 30 minutes	(B1) 10 mL Centrifuged SST Gold top	Aliquots (B1, B2, B3……..Bx). Serum aliquots stored for future testing
		Centrifuge for 20 minutes at 3000 rpm		
		Transfer 1.5 mL of supernatant into 3 × 1.8-mL cryovials		
		Affix barcode label		
		Store at −20°C		
		Ship on dry ice		
C (Blood)	2 × 4.5 mL purple top)(K2EDTA)	Do not process	(C1) 4.5 mL whole blood	Whole blood for DNA extraction
		Affix the barcode label		
		Store at −20°C		
		Ship on dry ice		
D (Blood)	4.5 mL Purple top (K3 EDTA)	Centrifuge for 20 minutes at 3000 rpm		Plasma fractions Buffy coat (0.5-mL cryovials) Red Blood Cells
		Transfer 1.5 mL plasma into 1 × 1.8-mL cryovial		
		Gently extract the buffy coat, transfer into 1 × 0.5-mL cryovial		
		Transfer 1.5 mL red blood cells into 1 × 1.8-mL cryovials		
		Affix barcode label		
E (Blood)	4.5 mL Blue top (Na citrate)	Centrifuge for 20 minutes at 3000 rpm	(E) Blue top plasma transfer tube	Plasma aliquots (E1, E2) Store for future analysis (e.g., TNF, fibrinogen)
		Transfer 1.5 mL of supernatant into × 1.8-mL cryovials		
		Affix barcode label		
		Store at −20°C		
		Storage within 1 hour		
F (Blood)	4.5 mL (light green top)	Centrifuge for 20 minutes at 3000 rpm		For lipid profile analysis
		Transfer 1.5 mL of supernatant into 3 × 1.8-mL cryovials		
		Affix barcode label		
		Store at −20°C		
G (Blood)	4.5 mL purple top (K3 EDTA)	Do not process		For HbA1c analysis
		Affix the barcode label		
		Do not store		
H (Urine)	Random Spot Urine (20-mL bottles)	Affix label. Do not aliquot. Store at −20°C and ship on dry ice	(U1) Urine creatinine and albumin.	Store for future analysis.

### Storage of blood fractions

Proper storage of samples for analysis is very crucial to ensure stability of the samples and accurate results of analysis anytime it is carried out.^23^ Fractions obtained after processing blood sample are initially stored locally in peripheral sites (at −20°C or −40°C) for up to 1°month and subsequently shipped to the Central Biorepository in Ibadan (−80°C). Central Laboratory specimens are stored in a manual defrost freezer until shipment to the Central Laboratory in Ibadan or Accra. Specimens stored in a freezer at −80°C or colder are shipped every 3 months, whereas specimens stored in a freezer warmer than −80°C are shipped every 4 weeks. The barcoded cryovials containing the samples are arranged into 10 × 10 freezer boxes. The cryovials containing the samples are arranged based on the different blood fractions. The barcodes on the cryovials are scanned to accurately display the samples' unique identification numbers on the excel sheet, which is saved on the computer dedicated to the project. The freezer boxes containing the cryovials are then arranged into each shelf of racks in the −80°C freezer until a shelf is filled.

### Daily temperature log

**FIG. 4.**
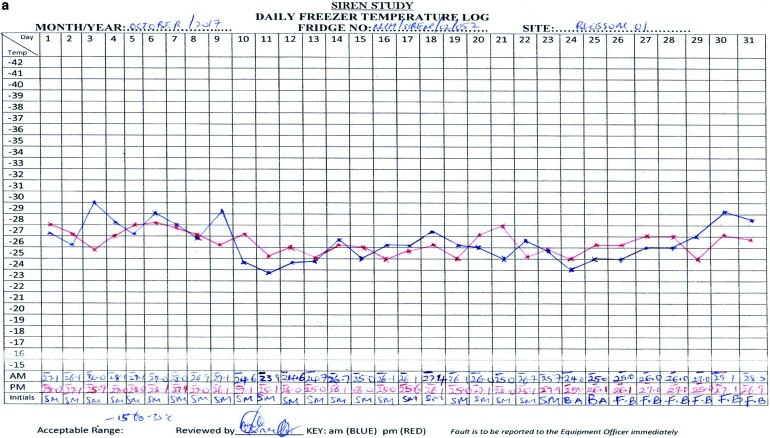

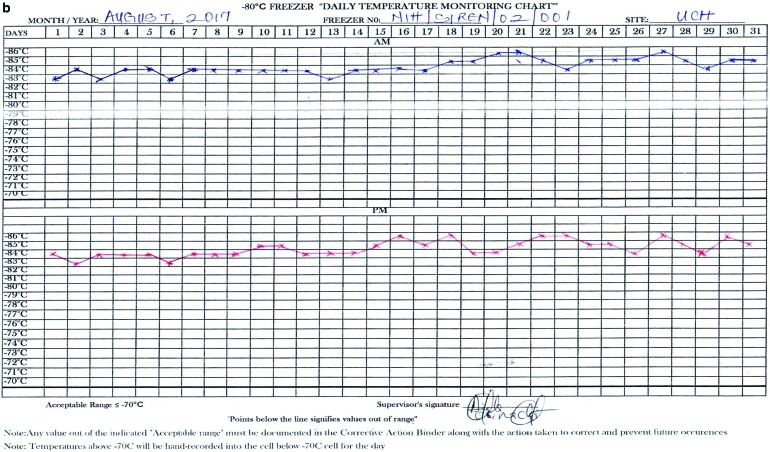
Stroke Investigative Research and Educational Network. (a) SIREN Temperature Chart −30°C. (b) SIREN Temperature Chart −80°C. Color images available online at www.liebertpub.com/bio

Daily temperature logs are kept to monitor the temperature of all freezers (including the −20°C, −30°C, −40°C, and −80°C) and to ensure that the freezers are working and within an acceptable range of temperatures (Fig. 4a, b).

### Limited sample analysis: determination of blood glucose level and lipid profile in SIREN subjects

Established cardiometabolic risk factors for stroke include diabetes mellitus and dyslipidemia. To establish the glycemic status of the subjects, fasting blood glucose and glycated hemoglobin levels are determined as well as fasting levels of cholesterol, triglycerides, and high- and low-density lipoproteins to establish dyslipidemia. Spot determinations of plasma glucose levels are carried out across all study sites using the ACCU-CHEK Active Blood Glucose Monitoring Device (Roche Diagnostics, GmBH, Germany), the principle of which is based on the reaction of blood glucose with the glucose dehydrogenase enzyme resulting in color changes, which the meter converts to numerical values. Values obtained in mg/dL are converted to mM.^24^

Glycated hemoglobin (HbA1c) levels are also determined on whole blood from all subjects within 24 hours of sample collection using the Clover A1c Test Cartridge System (Infopia Co. Ltd., Korea). The Clover A1c system uses the principle of boronate affinity chromatographic method for the determination of HbA1c in whole blood.^25^ Reagents in the system lyse red cells and bind hemoglobin, and also the boronate resins bind the cis-diols of glycated hemoglobin. These are measured separately within the system and the ratio of glycated hemoglobin to total hemoglobin is expressed as a percentage.

The fasting lipid profile of subjects is determined by quantitative determination of cholesterol, triglycerides, and HDL cholesterol using commercially available kits (Randox Laboratories Ltd. United Kingdom; Biolabo S.A., France), and the LDL cholesterol is calculated using the Friedewald equation.^26^ Cholesterol and triglycerides are determined using the enzymatic hydrolysis/colorimetric method, whereas HDL cholesterol is determined by precipitation method and the cholesterol fraction measured as described.^27,28^ Values obtained in mg/dL are converted to mM.^29^

To ensure equivalence across all sites, the SOP developed on the above laboratory tests are applied across all SIREN sites. The same brand of test equipment, reagents, including control sera, and test strips are procured and utilized across study sites as much as possible.

### DNA extraction

Twenty milliliters of whole blood is obtained using Vacutainer EDTA tubes, refrigerated at each study site and transferred to the Genomic Laboratory at Ibadan, Nigeria, the Molecular facilities at the Clinical Virology Laboratory, Department of Microbiology, University of Ghana Medical School, Accra, Ghana, and the Department of Medicine Research Laboratory, Komfo Anokye Teaching Hospital, Kumasi for processing.

Genomic DNA is extracted from whole blood using the Gentra Systems PUREGENE DNA Purification Kit (Qiagen Group) (see Supplementary Data on DNA Extraction Protocol).^30,31^ The DNA quality is ascertained using a NanoDrop spectrophotometer which measures the DNA concentrations at 260/280 nm and 260/230 nm, then determine the ratio to establish the quality of the DNA. In Ghana, extracted DNA samples in aliquots are shipped to the SIREN Biobank in Ibadan, Nigeria, on dry ice for long-term storage (at −80 degrees) after initial storage at local freezer temperature. This is followed by shipment of parts of the aliquots to the H3Africa I-HAB Biobanking Facility in Abuja, Nigeria, for storage and collaborating institutions for genomic analyses. All subjects' biosamples are given unique barcode identification numbers.

## Quality Control

**FIG. 5.**
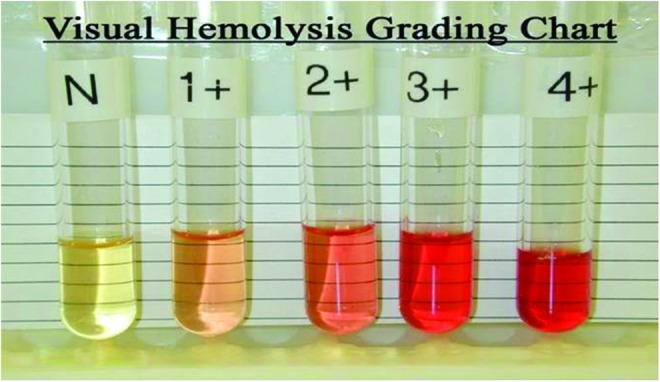
Visual grading for serum and plasma. Color images available online at www.liebertpub.com/bio

Each laboratory at the peripheral site has adopted the SOPs developed centrally for every laboratory and biorepository process involved in SIREN. This is in addition to the quality control/assurance practices in each center. Each site team of local principal investigator, laboratory scientist, and research coordinator work in synergy to ensure smooth implementation of the quality control/assurance standards. Subject preparation, sample collection, sample processing, and analyses are done by well-trained and highly skilled laboratory personnel. A visual grading system for hemolysis is employed as a quality control check for plasma and serum fractions from blood. Each serum or plasma sample is placed by the side of a chart showing the different degrees of hemolysis (Fig. 5). For the lipid profile assay, the quality control process of each peripheral site hospital is adopted, whereas quality control cartridges provided by the manufacturers for the Accu-Chek glucose strip and the clover cartridge system are used for plasma glucose and glycated hemoglobin quality control, respectively.

## Laboratory Information Management System

A LIMS is a software-based system with features that support a modern laboratory's operations, by allowing electronic management of samples and associated clinical data to improve laboratory efficiency.^32^ Freezerworks' Basic 7.2^®^ is the LIMS software used by the SIREN project for the management of the blood samples and fractions obtained from the study participants. Freezerworks is a product of Dataworks Development Inc., USA (Dataworks Development, Seattle, WA).^19,33^ Some of its features include sample tracking and freezer inventory management software. With a barcode scanner, the barcode label on each sample received from peripheral sites is scanned and the generated data are imported into the Freezerworks' database. Apart from the management of samples and associated data, Freezerworks allows additional data operations, such as audit management (fully track and maintain an audit trail), barcode handling (assign one or more data points to a barcode format, read, and extract information from a barcode), and facilitates access to specific data records and the person managing them.

The SIREN Biorepository Committee considered the following key factors in selecting a LIMS for the project: (a) usability and customizability, (b) cost and access to technical support services, (c) maintenance and associated cost, (d) robustness to handle large volumes of sample information, (e) security systems such as privileges, user roles, audit trail etc. and (f) support for barcode design and printing.^34^

Taking the above factors into consideration and the consideration for a standalone or shared LIMS across SIREN sites, the SIREN team chose a standalone Freezerworks V7.2. (Dataworks Inc., USA) since all biospecimens from peripheral sites were to be shipped to the central hub at Ibadan for long-term storage and management.

IT Support Structure: A combined set of the hardware (e.g., servers and computers), software (e.g., operating software), and network systems is required to deploy and support the LIMS. To ensure adequate IT infrastructural support with a fast processing speed and timely response to queries as the number of biospecimens grows in the biorepository, high-end laptops were dedicated to the LIMS. In addition, a dedicated external 1 terabyte hard disk for backing up the LIMS database was procured. In-house expertise for backing up and troubleshooting the LIMS was also acquired.

## Linkage to REDCap Database

The SIREN Project utilizes the Research Electronic Data Capture (REDCap),^35^ an electronic online database for storing the phenotype data from study participants. This database is hoisted on the server of the US collaborating institution, the Medical University of South Carolina (MUSC).The Electronic Data Interchange (EDI) operational features provided by the Freezerworks program enables the integration of data from the biorepository's LIMS with phenotype data on the REDCap database.

## Sample Shipment

Since human biospecimens are precious resources, adequate care is taken to preserve and maintain the integrity of these samples during the transportation process. To ensure an error-free shipment of the SIREN samples, proper preshipment preparations are made, including personnel training on shipping regulations and requirements for diagnostic specimens and infectious substances, consideration of the type and number of biospecimens, shipping time, distance, climate, method of transportation, and regulations. In addition, MTA import permits and requisitions for samples are secured as required. There are also email and telephone conversations with the intended consignee to finalize arrangements for an error-free process before the shipment is initiated.

Shipments are usually planned in such a way that the consignment is received by the consignee at least 2 days before a public holiday or the last working day of the week. Shipments are carried out if the courier company guarantees delivery the following day (for within country shipments) and can ensure the cold chain is maintained until the shipment is delivered to the recipient with adequate replenishment of dry ice. Before a shipment is scheduled for pickup, it is verified that all required shipping supplies are available such as outer shipping container/box, ice packs, dry ice, labels, and documents or prior arrangements are made with the courier company to provide all required shipping materials.

To initiate shipment, the samples are neatly arranged in the appropriate shipment containers using the triple packaging system and accompanying manifests (electronic and manual) are prepared.

The documents required for biological sample shipment include Custom invoice shipper's declaration, shipping log, MTA, shipper's waybill (to provide contact information and to declare nature of contents to customs and regulatory agencies), shipping manifest, and Import permit. To further ensure an error-free shipment, the choice of courier companies is based on such characteristics as reliability, experience with and ability to routinely ship human biological materials to national and international destinations, ability to provide online tracking of shipments, knowledge about relevant transportation regulations and permits, existence of established standardized paperwork accompanying shipments, efficient customer service ensuring that unforeseen delays and deviations are tracked and communicated to relevant personnel, customer service agents capable of troubleshooting and expediting shipments in accordance with temperature and time sensitivity of the samples, and willingness to “top-up” dry ice in the package in the event of a delay in transit.

The SIREN project has successfully shipped samples (DNA, blood fractions) to the Institute of Human Virology H3Africa Biorepository (I–HAB), Abuja, Nigeria and also to the University of Alabama, Birmingham, USA for genotyping (DNA). The samples shipped to I–HAB were validated to be of excellent quality (Table 5).

**Table 5.** Concentration and Purity of DNA Samples in SIREN Project

*Study*	*Concentration (ng/μL)*	*260/280 ratio*
SIREN Internal validation (*n* = 1999)	133.35 ± 100.34	1.82 ± 0.07
I–HAB External validation (*n* = 200)^a^	129.57 ± 82.17	1.84 ± 0.04

^a^ Samples were selected by systematic random sampling technique.

## Current Status of the SIREN Biorepository

The SIREN central hub is located in the Diagnostic and Research Laboratory of the Department of Medicine, College of Medicine, and University College Hospital, Ibadan. It has facilities, including two sets of ultra-low temperature (−80°C) freezers for long-term sample archiving and three sets of −30°C freezers for temporary sample storage before archiving in the −80°C freezers. All facilities are backed up with solar-powered uninterrupted power supply as shown in Figure 1. The biorepository currently has 3015 brain images, 92,950 blood fractions (serum, plasma, red blood cells, and buffy coat) accrued from 8450 recruited subjects and quantified and aliquoted good-quality DNA extracts from 6150 study subjects as at 14th November, 2017. These samples are currently distributed across all study sites, but ultimately will be moved to the growing biobanking facilities at the central site at the University College Hospital, Ibadan, Nigeria.

## Data and Biospecimen Access and Exploitation Plan

Data and biospecimens from SIREN will be made as widely and freely available as possible while safeguarding the privacy of participants, and protecting confidentiality and proprietary data in accordance with H3Africa consortium data sharing, access, and release policy, as well as the guidelines on data and biospecimen access policy of the H3Africa Consortium. External requests for SIREN data by researchers outside the H3Africa consortium will be processed by the H3Africa Data and Biospecimen Access Committee subject to the H3Africa Consortium data sharing, access and release policy and the SIREN data sharing plan. (https://h3africa.org/consortium/documents)

Access to biospecimens will require an MTA detailing the type of materials, the intended use of the samples, the location of storage outside Nigeria, duration of such storage, limitations on use, transfer, and termination of use of such materials subject to any law, regulations, and enactment in Nigeria. The MTA shall be signed by all parties involved in the research, including local and international principal investigators, heads of local institutions, research sponsors, and other relevant parties.

The different SIREN samples will be subjected to hypotheses-driven complementary “trans-omics” analyses. For instance, any genetic associations discovered at the level of genotyping DNA samples will be examined further by detecting its complementary biomarker in the serum and/or plasma or measuring relevant metabolites in the urine.

Governance of the SIREN Biobank is coordinated by the Study PI in charge of the biorepository under the supervision of the overall SIREN PI who in turn reports to an Advisory Committee. The SIREN Biorepository Committee is composed of the site-based laboratory coordinators, and the committee meets regularly by Skype to address issues as they arise. External oversight is provided by the H3Africa hub at the Institute of Human Virology, Abuja.

## Sustainability Plan

SIREN is in the process of securing additional future grants to invest in biobanking capacity building and securing more storage freezers. In addition, through our local Center for Genomic and Precision Medicine, we are working with our institutional leadership and building synergies with other researchers to upgrade our biobank to an institutional biorepository with robust support base. The project has enjoyed the administrative and infrastructural support of the federal and state governments in addition to the institutional support (College Research Innovation and Management Unit and the Center for Genomic and Precision Medicine, College of Medicine, University of Ibadan). Recently, the dividends of the SIREN project have been gaining central focus in the Oyo State Government's policy development plans. This will further position SIREN project arms, including the biobank for more support by the government.

## Lessons Learned and Future Directions

The journey of setting up the SIREN Biobank has been a great learning experience for the team through the challenges faced and innovative and pragmatic solutions devised to tackle each problem (Table 6). This experience typifies what occurs in a resource-constrained setting. This experience shows the possibility of establishing standard biobanks in low- and middle-income countries, particularly, in the context of a supportive collaborative network such as that provided by the Human Heredity and Health (H3) Africa Consortium, with relevant funding and technical support.

**Table 6.** Challenges and Solutions

*Challenges*	*Solutions*
Evacuation of samples from peripheral sites when prolonged power outage is experienced	Tested and trusted courier company to assist with immediate sample evacuation from peripheral site when the need arises
Inadequate sample storage space	Peripheral sites with access to −80°C freezer facilities secure temporary storage spaces
Refusal of study subjects to donate enough blood during recruitment	We persuade our study subjects by educating them on the purpose of the study and the free medical test they intend to benefit
Breakage of K2EDTA sample collection tubes	Replacement of K2EDTA tubes with plastic EDTA containers
Pressure on the field to package sample collection tubes to recruit subjects into the study	Prepacking of sample collection tubes before going to the field to recruit subjects into the study
Pressure to spin samples and also barcode cryovials while spinning samples in the centrifuge after a community engagement	Prebarcoding of cryovials before returning from a community engagement outreach

The SIREN Biobank represents an invaluable resource for future research with expanding genomic and trans-omic technologies. This will facilitate the inclusion of indigenous African samples in cutting-edge stroke genomics research.^36^ Given the unique characteristics of the African genome (high heterogeneity and low linkage disequilibrium), and higher stroke genetic heritability in African ancestry populations,^37^ the African genome is well placed for fine mapping of genetic regions associated with complex disorders, such as stroke, which have been previously identified in European ancestry populations.^36,38^ This approach has potential to deepen our understanding of the neurobiology of complex brain disorders and facilitate beneficial application of precision medicine in African ancestry and other global populations.^3,39^ Precision stroke medicine involves the acquisition of multiple datasets, including clinical phenotype, biological data (genomic, blood biomarkers), imaging data, and data integration (bioinformatic analysis, systems biology, and modeling) toward individualized diagnosis and treatment as well as prediction of recurrence risk, medication effects, procedural outcomes, and stroke recovery. Biological samples stored in a biobank provide resources to generate biological data (omics datasets), which contribute to the process of delivering tailored interventions.

Moving forward, it is vital to effectively engage African stroke patients and community members who have contributed precious biological materials to the SIREN Biobank. It is necessary to generate an appropriate evidence base for dealing with ethical, legal, and social issues of privacy, autonomy, identifiability, biorights, governance issues, and public understanding of stroke biobanking in the context of unique African culture, language, and belief systems.^40^

## Conclusion

The experience of SIREN in establishing its biobank shows that biobanking for brain disorders is feasible in Africa. It is now necessary to secure further public trust for the progress of biobank science and enhance public understanding of and participation in biobanking research activities in Africa. There are potential unique discoveries and research breakthroughs that will benefit African ancestry and other ancestral populations, since “*we are all Africans under the skin.”*^41^

## Supplementary Material

Supplemental data
