# Supplementary material for: Biobanking in a Challenging African Environment: Unique Experience from the SIREN Project
Source: Biopreserv Biobank. 2018 Jun 1;16(3):217–32. doi: 10.1089/bio.2017.0113 (PMC5995267; doi:10.1089/bio.2017.0113)
Supplement: Supplemental data [file Supp_Data.doc]

# Supplementary Data

# SIREN INFORMATION SHEET AND CONSENT FORM - CASE

IRB Research approval number: UI/EC/13/0105

# Title of Research: Stroke Investigative Research and Education Network (SIREN)

Principal Investigators: Dr. Mayowa O. Owolabi, MBBS, MSc, MD, FMCP.

Senior Lecturer, University of Ibadan

Dr. Bruce Ovbiagele, MD, MSc, MAS, FAAN.

Professor of Neurology

Medical University of South Carolina

Funded by: National Institutes of Health, USA.

You are being invited to participate in SIREN, a project designed to better understand the established and new environmental and genetic risk factors for stroke in populations of African ancestry. We hope that you will find this information helpful.

The University of Ibadan in collaboration with a network of other universities and research centers in Nigeria, Ghana, South Africa, UK, and US is conducting a research study (SIREN) on black populations of West Africa and the US, to find out more about how lifestyle, medical conditions (e.g. blood pressure and cholesterol), and genes act together to influence the development of stroke in people of black African origin. Our study is part of the Human Heredity and Health in Africa (“H3Africa”) project. We are asking everyone who comes to this hospital/clinic with acute stroke (within two weeks) to participate in this study. Approximately 3,000 patients with stroke and 3,000 people without stroke in Nigeria and Ghana will take part in this study. We hope to perform a much larger study in the future based on the results of this study.

**Why is SIREN being done?**

As you may know, stroke is the major cause of death and disability worldwide. We know that a person’s chance of getting a stroke is related to lifestyles such as eating habits, level of exercise, as well as exposure to tobacco smoke, blood pressure, and diabetes. Scientists have also found

that many genes may be linked to stroke and we expect they will find many more in the future. The purpose of SIREN is to learn about the role of these risk factors and genes for stroke in populations of African descent in West Africa and the US and the differences in the types of stroke people get. This will help decide if changing these risk factors can prevent stroke or affect the severity of stroke. It will also help explain the peculiarities of risk factors and stroke types among black people. This is the first and largest study of its kind and we hope that the information we get from the findings will benefit people who are predisposed to developing the disease condition.

Please ask us questions about anything you do not understand or if you would like more information, we are happy to explain this to you more than once.

## How long will this research last and how much of your time will be involved?

To meet up with our target sample size of 6,000 participants, the study will run for 4 years but your involvement will only last while you are admitted on the ward and for at least 1 month after the stroke episode. You may be contacted for follow-up afterwards if you wish to continue to participate.

## What is involved in participating in SIREN?

We will help you to fill out a questionnaire about your health, diet, exercise, and your use of tobacco, alcohol, stress, previous medical problems, medications, and your level of knowledge about stroke and other aspects of your health. The tests and analysis required of you are part of the harmless routine procedures needed for treating your disease condition and they include: evaluation of your blood pressure, heart rate, height, weight, waist and hip circumference, brain scan (CT-Scan/ MRI), ECG, Echocardiography, and duplex ultrasound of the vessels supplying your brain In addition, we will need to withdraw about 35mL of blood (this is the same as 3 tablespoons) part of which will be used to test for lipids, sugar levels, and genetic analysis while the other will be stored for future analyses. We will also collect about 20mL of your urine for further analysis.

We will need less than 5 minutes to take the blood sample from a vein in your arm. There will be no medicines to take and no experimental treatments to undergo in SIREN. Nothing else is required. The only genetic testing on your blood sample will be for conditions associated with stroke.

## How will information about me be kept private?

To protect your privacy, we will replace your name with a code (numbers, letters, or both). We will separate your name and any other information that points to you from your blood sample and your data. We will keep files that link your name to the code number in a locked cabinet. Only permitted members of the research team will have access to these files. No one who reads or hears a report about SIREN will be able to identify you because, before any facts are given out, we combine your facts with those of other people in the study. Your name or other facts that might point to you will not appear when we present this study or publish its results.

A goal of H3Africa is to create a way for researchers to share and learn from each other especially within Africa. One of the best ways to do this is for scientists to share research data. We would like your permission to share your health history, laboratory test results, and your genomic information. When we share this information, people will not know your name. Other investigators who want to use your data (without your name) will need to first ask for permission from the central repository. They will need to agree only to use the data for a specific scientific research. There is a small chance that your data together with the data of many other people could be used to develop new treatments, which might be beneficial to you or other people. If this happens, it is unlikely that the proceeds will be shared with you.

## What will happen to the samples?

Your blood samples will be stored in a locked freezer in our laboratory and your personal information on a secure computer. Furthermore, part of your sample will be stored together with the other samples that people have given from all over Africa as part of a big collection that is called a “biobank”. The biobank will be somewhere on the African continent where your sample will be stored under lock and key. Researchers from all over the world can ask to use these samples for their research. Although the study you are being asked to participate in is related to stroke, other scientists may like to use your sample to study other diseases. The samples will not be sold, but investigators may develop products based on studying your samples. You can choose not to have your sample shared with other researchers and still be part of SIREN. You have your chance of stating this on the SIREN Consent form.

**Voluntary nature of participation and right to withdrawal**

## It is a personal decision whether you take part in the study. You can say “Yes” and join the study or you can also say “No” you do not want to join. If you participate in the study, you can change your mind later and decide that you do not want to participate anymore and you do not want your blood to be used in this study. Please let us know and we will destroy the sample. If your sample has already been tested at the time you change your mind, your results and other data may have already been shared with investigators. In that case we will not be able to destroy these data. Your data can be removed from the central repository, however, that means that no additional researchers get your data.

Whether you decide to join or not to join the study, the way we look after you in this clinic will be the same. It is your decision whether to be in the study or not.

**Potential Risks associated with the research**

We want to tell you of the possible risk associated with this study. Most of the time when we take blood it is safe, but sometimes when we take the blood sample, people feel a bit faint or very rarely get an infection. You may also get a slight bruise where we took the blood, if it does happen you will be treated immediately without extra costs.

**Potential benefits associated with the research**

This study may not directly help you (or your children) to get better, but we hope it will benefit others in the future. What we are trying to do is very complex and could take a long time. Whether you decide to join this study or not will not affect your treatment in the clinic. Your decision to join is of your own free will.

**How will I find out the results of the study?**

We plan to share some of the results of tests conducted during this project (e.g duplex ultrasound, blood sugar) that may contribute to your medical care with you. We will attempt to input relevant results into your medical records.

This study will take a while before it is concluded and when it is finished observations and discoveries will be shared with other health professional through publications. However, if you have any questions, you could ask your doctor.

## Are any costs or payments involved?

It does not cost you anything to provide a blood sample for SIREN and you will not be charged for any tests required specifically for this research only. Although, you will not receive any monetary gratification for participating in this study, you will enjoy excellent clinical care and have access to communicate issues about your treatment with the clinicians working with the team of investigators. We will do everything possible to avoid any degree or form or injury while undergoing clinical assessment or giving a blood sample, nonetheless we will provide adequate treatment in the very unlikely event that this occurs.

# SIREN CONSENT FORM – Case

Principal Investigators: Dr. Mayowa O. Owolabi MBBS, MSc, MD, FMCP.

Senior Lecturer, University of Ibadan

Dr. Bruce Ovbiagele, MD, MSc, MAS, FAAN.

Professor of Neurology

Medical University of South Carolina

Funded by: National Institutes of Health, USA.

This research, which is part of the Human Heredity and Health in Africa (“H3Africa”) project, has been approved by the University of Ibadan/University College Hospital Ethics Committee, Ibadan and the Chairman of this Committee can be contacted at IMRAT, UCH, Ibadan. Email: academic.uiuchirc@yahoo.com

In addition, if you have any question about your participation in this research, you can contact the principal investigator Dr. Mayowa Owolabi, Department of Medicine, University College Hospital, Ibadan.

## Consents and Signature

I agree to participate in SIREN. I understand that I will fill out a brief survey about my health, lifestyle, food habits, medications, and my knowledge about stroke. I will have about 35mL of one blood sample drawn and about 20mL of urine collected. The tests to be carried out on my samples will include cholesterol, glucose, and genetic factors. I will also have my blood pressure and heart rate taken, my height, weight, waist, and hip circumference measured. My medical record will be accessed for my medical and treatment information. My only discomfort will be related to the needle prick for drawing the blood, and the most common adverse effect is bruising around the vein from where the blood sample is drawn.

I understand that my participation in the study is voluntary. All information gathered for this study is strictly confidential and will not be used to create financial profit. I will not be identified in any published report. I am free to refuse to participate or withdraw from the study at any time without jeopardizing my future care. I have been given a chance to ask questions and feel that all of my questions have been answered.

I know that giving a blood sample for this study is my choice. I understand that my individual results will not be given to me. I will receive a signed copy of this form. I have read the part of the Information Sheet about storing my blood sample for future research. My choice about having my blood sample stored and used for research under the conditions described is: (please check ONE box)

- I agree to participate in SIREN but do not want my sample to be shared with other investigators outside SIREN.
- I agree to participate in SIREN and my sample can be shared with other investigators within H3 Africa only for research in a related field (eg cardiovascular research)
- I agree to participate in SIREN and my sample can be shared with other investigators in any other research field

__________________________________________

Name of Participant

_______________________________________________

Name of Authorized Representative (if applicable)

_______________________________________________ ______________________

Signature of Participant or Authorized Representative Date

Consent form administered and explained in person by:

_____________________________________

Name and title

_____________________________________ ____________________

Signature Date

# In my judgment, the participant is voluntarily and knowingly giving informed consent and possesses the legal capacity to give informed consent to participate in this research study.

# _ ______________­­________________________

# Name of Investigator

# __________________________________________ ______________________

# Signature of Investigator Date

PLEASE KEEP A COPY OF THE SIGNED INFORMED CONSENT.

# SIREN INFORMATION SHEET AND CONSENT FORM - CONTROL

IRB Research approval number: UI/EC/13/0105

# Title of Research: Stroke Investigative Research and Education Network (SIREN)

Principal Investigators: Dr. Mayowa O. Owolabi, MBBS, MSc, MD, FMCP.

Senior Lecturer, University of Ibadan

Dr. Bruce Ovbiagele, MD, MSc, MAS, FAAN.

Professor of Neurology

Medical University of South Carolina

Funded by: National Institutes of Health, USA.

You are being invited to participate in SIREN, a project designed to better understand the established and new environmental and genetic risk factors for stroke in populations of African ancestry. We hope that you will find this information helpful.

The University of Ibadan in collaboration with a network of other universities and research centers in Nigeria, Ghana, South Africa, UK, and US is conducting a research study (SIREN) on black populations of West Africa and the US, to find out more about how lifestyle, medical conditions (e.g. blood pressure and cholesterol), and genes act together to influence the development of stroke in people of black African origin. Our study is part of the Human Heredity and Health in Africa (“H3Africa”) project. We are also people without stroke to participate in this study. Approximately 3,000 patients with stroke and 3,000 people without stroke in Nigeria and Ghana will take part in this study. We hope to perform a much larger study in the future based on the results of this study.

**Why is SIREN being done?**

As you may know, stroke is the major cause of death and disability worldwide. We know that a person’s chance of getting a stroke is related to lifestyles such as eating habits, level of exercise as well as exposure to tobacco smoke, blood pressure, and diabetes. Scientists have also found

that many genes may be linked to stroke and we expect they will find many more in the future. The purpose of SIREN is to learn about the role of these risk factors and genes for stroke in populations of African descent in West Africa and the US and the differences in the types of stroke people get. This will help decide if changing these risk factors can prevent stroke or affect the severity of stroke. It will also help explain the peculiarities of risk factors and stroke types among black people. This is the first and largest study of its kind and we hope that the information we get from the findings will benefit people who are predisposed to developing the disease condition.

Please ask us questions about anything you do not understand or if you would like more information, we are happy to explain this to you more than once.

## How long will this research last and how much of your time will be involved?

To meet up with our target sample size of 6,000 participants, the study will run for 4 years, but your involvement will initially last an hour at most at the clinic visit, where we will collect the information from you. You will then be requested to participate in further tests (e.g ECG, blood tests) on the same day or at another date.

## What is involved in participating in SIREN?

We will help you to fill out a questionnaire about your health, diet, exercise, and your use of tobacco, alcohol, stress, previous medical problems, medications, and your level of knowledge about stroke and other aspects of your health. The tests and analysis required of you are harmless procedures and they include: evaluation of your blood pressure, heart rate, height, weight, waist, hip circumference, and ECG. In addition, we will need to withdraw some blood from you part of which will be used to test for lipids, sugar levels, and genetic analysis, while the other will be stored for future analyses. We will also collect urine for further analysis.

We will need less than 5 minutes to take the blood sample from a vein in your arm. There will be no medicines to take and no experimental treatments to undergo in SIREN. Nothing else is required. The only genetic testing on your blood sample will be for conditions associated with stroke.

## How will information about me be kept private?

If you decide to participate, we will assign you a code number. We will separate your name and any other information that points to you from your blood sample and your survey. We will keep files that link your name to the code number in a locked cabinet. Only the study investigators will have access to these files. No one who reads or hears a report about SIREN will be able to identify you because, before any facts are given out, we combine your facts with those of other people in the study. Your name or other facts that might point to you will not appear when we present this study or publish its results.

## How will information about me be kept private?

To protect your privacy, we will replace your name with a code (numbers, letters or both). We will separate your name and any other information that points to you from your blood sample and your data. We will keep files that link your name to the code number in a locked cabinet. Only permitted members of the research team will have access to these files. No one who reads or hears a report about SIREN will be able to identify you because, before any facts are given out, we combine your facts with those of other people in the study. Your name or other facts that might point to you will not appear when we present this study or publish its results.

A goal of H3Africa is to create a way for researchers to share and learn from each other especially within Africa. One of the best ways to do this is for scientists to share research data. We would like your permission to share your health history, laboratory test results and your genomic information. When we share this information, people will not know your name. Other investigators who want to use your data (without your name) will need to first ask for permission from the central repository. They will need to agree only to use the data for a specific scientific research. There is a small chance that your data together with the data of many other people could be used to develop new treatments which might be beneficial to you or other people. If this happens, it is unlikely that the proceeds will be shared with you.

## What will happen to the samples?

Your blood samples will be stored in a locked freezer in our laboratory and your personal information on a secure computer. Furthermore, part of your sample will be stored together with the other samples that people have given from all over Africa as part of a big collection that is called a “biobank”. The biobank will be somewhere on the African continent where your sample will be stored under lock and key. Researchers from all over the world can ask to use these samples for their research. Although the study you are being asked to participate in is related to stroke, other scientists may like to use your sample to study other diseases. The samples will not be sold, but investigators may develop products based on studying your samples. You can choose not to have your sample shared with other researchers and still be part of SIREN. You have your chance of stating this on the SIREN Consent form.

**Voluntary nature of participation and right to withdrawal**

## It is a personal decision whether you take part in the study. You can say “Yes” and join the study or you can also say “No” you do not want to join. If you participate in the study, you can change your mind later and decide that you do not want to participate anymore and you do not want your blood to be used in this study. Please let us know and we will destroy the sample. If your sample has already been tested at the time you change your mind, your results and other data may have already been shared with investigators. In that case we will not be able to destroy these data. Your data can be removed from the central repository, however, that means that no additional researchers get your data.

Whether you decide to join or not to join the study, the way we look after you in this clinic will be the same. It is your decision whether to be in the study or not.

**Potential Risks associated with the research**

We want to tell you of the possible risk associated with this study. Most of the time when we take blood it is safe, but sometimes when we take the blood sample, people feel a bit faint or very rarely get an infection. You may also get a slight bruise where we took the blood, if it does happen you will be treated immediately without extra costs.

**Potential benefits associated with the research**

This study may help us to discover conditions that may increase your risk of developing stroke. At your request, we can share this information with your doctor so they can help you prevent stroke and other diseases.

**How will I find out the results of the study?**

We plan to share some of the results of tests conducted during this project (e.g duplex ultrasound, blood sugar) that may contribute to your medical care with you. We will attempt to input relevant results into your medical records.

This study will take a while before it is concluded and when it is finished observations and discoveries will be shared with other health professionals through publications. However, if you have any questions, you could ask your doctor.

## Are any costs or payments involved?

It does not cost you anything to provide a blood sample for SIREN and you will not be charged for any tests required specifically for this research only. Although, you will not receive any monetary gratification for participating in this study, you will enjoy excellent clinical care and have access to communicate issues about your health with the clinicians working with the team of investigators. We will do everything possible to avoid any degree or form or injury while undergoing clinical assessment or giving a blood sample, nonetheless we will provide adequate treatment in the very unlikely event that this occurs.

# SIREN CONSENT FORM – Control

Principal Investigators: Dr. Mayowa O. Owolabi MBBS, MSc, MD, FMCP.

Senior Lecturer, University of Ibadan

Dr. Bruce Ovbiagele, MD, MSc, MAS, FAAN.

Professor of Neurology

Medical University of South Carolina

Funded by: National Institutes of Health, USA.

This research which is part of the Human Heredity and Health in Africa (“H3Africa”) project has been approved by the University of Ibadan/University College Hospital Ethics Committee, Ibadan and the Chairman of this Committee can be contacted at IMRAT, UCH, Ibadan. Email: academic.uiuchirc@yahoo.com

In addition, if you have any question about your participation in this research, you can contact the principal investigator Dr. Mayowa Owolabi, Department of Medicine, University College Hospital, Ibadan.

## Consents and Signature

I agree to participate in SIREN. I understand that I will provide information for filling out a brief survey about my health, lifestyle, food habits, medications, and my knowledge about stroke. I will have blood samples drawn, and urine sample collected. The tests to be carried out on my samples will include cholesterol, glucose and genetic factors. I will also have my blood pressure and heart rate taken, my height, weight, waist, and hip circumference measured. My only discomfort will be related to the needle prick for drawing the blood, and the most common adverse effect is bruising around the vein from where the blood sample is drawn.

I understand that my participation in the study is voluntary. All information gathered for this study is strictly confidential and will not be used to create financial profit. I will not be identified in any published report. I am free to refuse to participate or withdraw from the study at any time without jeopardizing my future care. I have been given a chance to ask questions and feel that all of my questions have been answered.

I know that giving a blood sample for this study is my choice. I understand that my individual results will not be given to me. I will receive a signed copy of this form. I have read the part of the Information Sheet about storing my blood sample for future research. My choice about having my blood sample stored and used for research under the conditions described is: (please check ONE box)

- I agree to participate in SIREN but do not want my sample to be shared with other investigators outside SIREN.
- I agree to participate in SIREN and my sample can be shared with other investigators within H3 Africa only for research in a related field (e.g. cardiovascular research)
- I agree to participate in SIREN and my sample can be shared with other investigators in any other research field

__________________________________________

Name of Participant

_______________________________________________

Name of Authorized Representative (if applicable)

_______________________________________________ ______________________

Signature of Participant or Authorized Representative Date

Consent form administered and explained in person by:

_____________________________________

Name and title

_____________________________________ ____________________

Signature Date

# In my judgment, the participant is voluntarily and knowingly giving informed consent and possesses the legal capacity to give informed consent to participate in this research study.

# _ ______________­­________________________

# Name of Investigator

# __________________________________________ ______________________

# Signature of Investigator Date

PLEASE KEEP A COPY OF THE SIGNED INFORMED CONSENT.
